# Immunotherapy for Patients with Advanced Urothelial Cancer: Current Evidence and Future Perspectives

**DOI:** 10.1155/2017/5618174

**Published:** 2017-06-07

**Authors:** Clizia Zichi, Marcello Tucci, Gianmarco Leone, Consuelo Buttigliero, Francesca Vignani, Daniele Pignataro, Giorgio V. Scagliotti, Massimo Di Maio

**Affiliations:** ^1^Department of Oncology, University of Turin, Division of Medical Oncology, San Luigi Gonzaga Hospital, Regione Gonzole 10, Orbassano, 10043 Turin, Italy; ^2^Department of Oncology, University of Turin, Division of Medical Oncology, Ordine Mauriziano Hospital, Via Magellano 1, 10028 Turin, Italy

## Abstract

In recent years, immunotherapy has produced encouraging results in a rapidly increasing number of solid tumors. The responsiveness of bladder cancer to immunotherapy was first established in nonmuscle invasive disease in 1976 with intravesical instillations of bacillus Calmette-Guérin (BCG). Very recently immune checkpoint inhibitors demonstrated good activity and significant efficacy in metastatic disease. In particular the best results were obtained with programmed death-ligand-1 (PD-L1) and programmed death-1 (PD-1) inhibitors, but many other immune checkpoint inhibitors, including anti-cytotoxic T-lymphocyte-associated protein-4 (CTLA-4) antibodies, are currently under investigation in several trials. Simultaneously other therapeutic strategies which recruit an adaptive immune response against tumoral antigens or employ externally manipulated tumor infiltrating lymphocytes might change the natural history of bladder cancer in the near future. This review describes the rationale for the use of immunotherapy in bladder cancer and discusses recent and ongoing clinical trials with checkpoint inhibitors and other novel immunotherapy agents.

## 1. Introduction

As well documented by a large body of research, tumor cells are able to avoid control and destruction by the immune system using a range of complex and often overlapping mechanisms that lead to disruption of key components involved in the effective antitumor response [1–4]. Immune system should recognize and eliminate tumor cells that can avoid this immune response by disrupting antigen presentation, either through downregulation of major histocompatibility complex (MHC) class I molecules or by disabling antigen-processing machinery. Alternatively, or in addition, tumors can be able to suppress the immune system by a disruption of molecular pathways involved in controlling T-cell inhibition and activation or by recruiting immunosuppressive cell types, such as regulatory T-cells (Tregs) and myeloid-derived suppressor cells. Another mechanism that tumor cells may use in order to suppress immune activity is the release of factors, including adenosine and prostaglandin E2 and the enzyme indoleamine 2,3-dioxygenase (IDO) [3].

The robust progress in the understanding of these tumor immune-evasion strategies has resulted in the evaluation of various approaches to target and harness the patient's immune system directly to kill tumor cells. Consequently, in recent years, new generation of immunotherapy has produced relevant results in a rapidly increasing number of solid tumors. With the exception of the therapeutic vaccine sipuleucel-T that was approved for the treatment of prostate cancer in 2010, all these practice-changing results have been obtained with immune checkpoint inhibitors. Two major classes of drugs have been tested: anti-cytotoxic T-lymphocyte-associated protein (CTLA)-4 antibodies and anti-programmed death-1 (PD-1) or anti-programmed death-ligand-1 (PD-L1) antibodies. Starting from melanoma, these drugs have produced positive results in many solid tumors. Differently from classical chemotherapy and from the majority of molecularly targeted agents that act by directly targeting tumor cells, all the immune checkpoint inhibitors act by targeting the patient's immune system against tumor cells.

First important results have been obtained with ipilimumab in patients affected by malignant melanoma [5, 6]. Subsequently, also nivolumab and pembrolizumab demonstrated efficacy in these patients [7–9].

Following the results obtained in patients with malignant melanoma, immune checkpoint inhibitors have produced clear evidence of efficacy, within randomized controlled trials, in the treatment of patients with advanced non-small-cell lung cancer (NSCLC). Namely, in patients who have failed first-line platinum-based chemotherapy, nivolumab, pembrolizumab, and atezolizumab, all given as single agents, demonstrated an improvement in overall survival compared to docetaxel [10–13]. In addition, pembrolizumab has also shown superiority compared to platinum-based chemotherapy, when given as first-line in a population of advanced NSCLC patients, selected for the high expression of PD-L1 in tumor cells [14].

Nivolumab has also been approved for the second-line treatment of advanced renal cell cancer, following the results of a randomized phase III trial showing an improvement in overall survival compared to everolimus [15].

Furthermore, the list of other solid tumors where immune checkpoint inhibitors have already produced evidence of activity and efficacy and where these drugs are currently under investigation is long.

## 2. Rationale for Immunotherapy in Urothelial Cancer

The efficacy of immunotherapy in bladder cancer was first established in 1976 when Morales et al. proved for the first time that intravesical instillations of bacillus Calmette-Guérin (BCG) were efficient in preventing recurrences of high-risk nonmuscle invasive urothelial bladder cancer and in treating carcinoma in situ [16]. Although the mechanism of action of BCG is not yet clear even after forty years from the first evidence, it seems to stimulate a cytotoxic response trough the combination of antigenic fragments, processed by bladder cancer cells, with the histocompatibility complex on the tumor cells surface [17].

After this initial success, many other attempts have been made to take advantage of directing T-cells against bladder cancer cells both in the localized and advanced disease, using activating cytokines such as interleukin- (IL-) 2 and interferon- (IFN-) alfa-2B [18, 19]. These drugs have shown limited benefits in achieving disease control.

A turning point took place on the second decade of this century when immune checkpoint inhibitors arrived on the scene. Contrary to the previous strategy this new class of monoclonal antibodies aims to reduce inhibitory signaling instead of directly stimulating T-cells.

The first receptor to be targeted was CTLA-4, a molecule expressed on activated CD4 and CD8 T-cells. CTLA-4 competes with CD28 for the interaction with the costimulatory CD80-CD86 molecules on antigen presenting cells (APCs). While the latter interaction promotes T-cells activation and effector functions, CTLA-4-CD80/86 inhibits T-cell activation in lymphoid tissues [20]. Two monoclonal antibodies targeting CTLA-4 have been developed: ipilimumab and tremelimumab, whose effect is to shift T-cell equilibrium toward activation.

It has been further observed that tumor cells might evade immune system surveillance through the interaction between PD-L1 and PD-L2 with their receptor PD-1, which is expressed on CD4 and CD8 T-cells, Tregs, B-cells, and natural killer (NK) cells. Acting directly among tumor microenvironment, drugs directed against either PD-1 or PD-L1 are usually characterized by lower adverse effects than CTLA-4 inhibitors [21].

Furthermore, many other immune checkpoint receptors are currently under investigation in several trials, as potential therapeutic targets. Simultaneously other therapeutic strategies which recruit an adaptive immune response against tumoral antigens might change the natural history of bladder cancer in the near future [20].

Bladder cancer usually shows some biological features that have been associated with better response to immunotherapies. First of all, an adaptive immune response against cancer cells requires the presence tumor antigens endowed with a good immunogenicity. More the mutation board, more likely this kind of antigens is expressed in tumor microenvironment. Bladder cancer is often characterized by a high mutation load. Moreover PD-L1 expression on the surface of tumor cells has been correlated with a higher-stage, suggesting good response to PD1/PD-L1 antagonist, although the results of different trials did not observe the association between PD-L1 expression and tumor response rate [22].

Indeed, the major challenge that is going to be faced in the next years is to find predictive factors granted by greater sensibility and specificity.

## 3. PD-L1 Inhibitors

### 3.1. Atezolizumab

Atezolizumab is an engineered human monoclonal antibody against PD-L1, able to inhibit the interaction between PD-L1 and its receptor PD-1. A multicentre, nonrandomized, phase II trial (IMVigor 210) evaluated the efficacy and safety profile of intravenous atezolizumab (given every three weeks at the dose of 1200 mg) in two different cohorts of locally advanced or metastatic urothelial carcinoma: cohort A included treatment naïve patients, ineligible for cisplatin; cohort B included patients progressing during or after platinum-based chemotherapy. PD-L1 expression on tumor infiltrating immune cells was assessed prospectively by immunohistochemistry. On the basis of PD-L1 expression, patients were categorized in three subgroups: IC0 (<1%), IC1 (≥1% but <5%), and IC2/3 (≥5%).

In cohort B, among the 310 evaluable patients overall response rate (ORR) was 15% (95% CI, 11–20) with 5% of complete responses. High levels of PD-L1 expression were associated with better ORR (27%; 95% CI, 19–37). After a median follow-up of 11.7 months, the median duration of response has not yet been reached, and durable responses have been recorded also in patients with poor prognostic features [23] (Table 1). Median overall survival was 11.4 months in patients in the IC2/3 group, 8.8 months in the IC1/2/3 group, and 7.9 months in the whole cohort of patients.

Due to these positive results, Food and Drug Administration (FDA) approved in May 2016 atezolizumab for the treatment of patients with locally advanced or metastatic urothelial carcinoma progressing during or following platinum-containing chemotherapy or within 12 months of either adjuvant or neoadjuvant platinum chemotherapy. The recommended dose is 1200 mg, given as an intravenous infusion every three weeks.

As for the cohort A of patients who were not eligible for cisplatin (*n* = 123), the ORR was 23% (95% CI, 16–31) in all patients, with slight but not statistically significant differences among PD-L1 subgroups. ORR was 28% (95% CI; 14–47) in patients with high PD-L1 expression and 21% (95% CI; 9–36) in patients PD-L1 negative. After a 17.2 months median follow-up duration, median overall survival (OS) was 15.9 months (95% CI; 10.4 to not estimable) in all patients [24] (Table 1).

In the attempt of identifying predictive factors of activity and efficacy of atezolizumab, in addition to PD-L1 determination, authors evaluated also Cancer Genome Atlas gene expression and mutation load. In both cohorts, responses were more frequent in the Luminal II subtype and in patients with higher mutation load, irrespective of PD-L1 expression. Moreover, in cohort B, PD-L1 expression and responses to atezolizumab were most closely associated with immune activation gene expression (e.g., interferon-*γ*-inducible T-helper-1-type chemokines: CXCL9 and CXCL10) and CD8 T-cell infiltration [23, 24].

Treatment with atezolizumab was well tolerated in both cohorts, with serious adverse events (AEs) occurring in 15-16% of patients, and only a treatment-related death for sepsis occurred in cohort A [23, 24].

A phase 3 trial is evaluating the efficacy of atezolizumab compared to second-line chemotherapy in patients with locally advanced or metastatic urothelial carcinoma progressing to platinum-based treatment; furthermore, several studies are ongoing investigating atezolizumab monotherapy or in combination with chemotherapy or other immunological agents in different stages of disease (Table 2).

### 3.2. Durvalumab

Durvalumab (MEDI4736) is a selective, high-affinity, human monoclonal antibody directed against PD-L1. A phase I/II multicentre dose escalation and dose-expansion study is ongoing in patients with advanced solid tumors, to evaluate safety, tolerability, and antitumor activity of durvalumab monotherapy. In June 2016, Massard et al. published first results about patients with urothelial carcinoma progressing on or ineligible for cisplatin-based therapy (*n* = 61). Durvalumab, at the dose of 10 mg/kg, was administered intravenously every two weeks, for up to twelve months. Patients were categorized on the basis of PD-L1 expression, assessed either on tumor cells or on immune cells (adopting a cutoff of 25%). Among the 42 patients evaluable for response, the ORR was 31% (95% CI, 17.6–47.1). A greater antitumor activity was observed in the PD-L1 positive subgroup (46.4%; 95% CI, 27.5–66.1); in the PD-L1 negative patients the ORR was 0% (95% CI, 0.0–23.2). At the time of analysis, responses were ongoing in 12 of 13 patients with a median duration of response not yet reached (range: 4.1 to 49.3 weeks). Treatment tolerance was optimal; serious AEs occurred in 4.9% of patients, with no treatment-related deaths [25] (Table 1).

An update of this study has been presented at 2017 ASCO Genitourinary Cancer Symposium. Efficacy analysis included 103 patients with a median follow-up of 7.3 months. The ORR was 20.4% (13.1–29.5) in the overall population and 29.5% (18.5–42.6) in the PD-L1 positive subgroup versus 7.7% (1.6–20.9) in the PD-L1 negative patients [26].

In February 2016 the FDA granted a breakthrough therapy designation to durvalumab as a treatment for PD-L1-positive inoperable or metastatic urothelial bladder cancer patients progressing on platinum-based treatment.

Several trials are ongoing in urothelial carcinoma patients investigating activity of durvalumab, alone or in combination with the anti-CTLA4 tremelimumab (Table 2).

### 3.3. Avelumab

Avelumab is a fully human anti-PD-L1 monoclonal antibody. A large phase Ib trial is ongoing, investigating safety, tolerability, and clinical activity of avelumab in patients with locally advanced or metastatic solid tumors, including patients with urothelial carcinoma whose disease progressed after platinum-based chemotherapy or who were platinum ineligible. Avelumab showed preliminary safety and efficacy in a cohort of 44 patients [27] (Table 1), so an additional cohort of 129 eligible urothelial carcinoma patients was enrolled and received avelumab, 10 mg/kg, every two weeks until confirmed progression, unacceptable toxicity, or withdrawal. Preliminary data about 109 patients with at least four months of follow-up were presented at 2016 ESMO Congress: confirmed ORR was 16.5% (95% CI, 10.1–24.8), with 3 complete and 15 partial responses. PFS rate at 12 weeks was 35.6 (95% CI; 26.5–44.7). Treatment was well tolerated; grade 3-4 treatment-related adverse events occurred in 9% of patients; and pneumonitis resulted in one treatment-related death [28]. An update of this study was reported at 2017 ASCO Genitourinary Cancer Symposium. Data were available in 153/241 patients with at least six months of follow-up: ORR was 17.6% (95% CI, 12.0–24.6), 88.9% of responses were ongoing at the time of analysis, and median OS was 7.0 months (95% CI, 5.6–11.1). Based on a ≥5% PD-L1 expression cutoff assessed prospectively on tumor cells, ORR was significantly higher in PD-L1 positive patients (25.0%; 95% CI, 14.4–38.4) compared with PD-L1 negative subgroup (14.7%; 95% CI, 7.6–24.7; *p* = 0.178). Treatment was well tolerated, with only 7.5% grade ≥ 3 treatment-related AEs [29].

A randomized, open-label phase 3 trial of avelumab + best supportive care (BSC) versus BSC alone as maintenance therapy after first-line platinum-based chemotherapy is ongoing in patients with advanced urothelial cancer (Table 2).

## 4. PD-1 Inhibitors

### 4.1. Nivolumab

Nivolumab is a fully human anti-PD-1 monoclonal antibody, currently approved for the treatment for different malignancies, as front-line (melanoma) or second-line monotherapy (NSCLC, renal cell cancer) or in combination with ipilimumab (melanoma). An ongoing open-label, two-stage, multiarm, phase I/II trial, Checkmate 032, is evaluating safety and activity of nivolumab alone or in combination with ipilimumab in subjects with advanced or metastatic solid tumors. First results about a cohort of patients with advanced urothelial carcinoma, who progressed during or after platinum-based chemotherapy, treated with nivolumab alone (3 mg/kg intravenously every two weeks), were published in October 2016. Eligible patients were enrolled regardless of tumor cells PD-L1 expression that was assessed retrospectively in pretreatment tumor biopsy specimens collected within three months before treatment start. A confirmed ORR was achieved in 24.4% (95% CI, 15.3–35.4) of 78 patients treated with nivolumab monotherapy, regardless of PD-L1 tumor expression. There was no difference in the ORR between patients with PD-L1 expression lower than 1% (26.2%) and patients with PD-L1 expression ≥ 1% (24.0%). However median OS was over 16.2 months in PD-L1 positive tumors and 9.9 months in PD-L1 negative ones [30] (Table 1).

These data were recently confirmed by positive results of phase II study, Checkmate 275, evaluating activity and safety of nivolumab in 270 patients with metastatic bladder cancer progressing during or after first-line platinum-based chemotherapy. Confirmed ORR was 19.6% (95% CI, 15.0–24.9) for all patients, 28.4% for patients with PD-L1 expression of 5% or greater, 23.8% for patients with PD-L1 expression of 1% or greater, and 16.1% for patients with PD-L1 expression of less than 1%. After a median follow-up equal to 7 months, 24.4% of patients were still on treatment. Median OS was 8.74 months in the whole study population; in the subgroup of patients expressing PD-L1 ≥ 1% on tumor cells median OS was 11.3 months, while in PD-L1 negative patients it was 5.95 months. Cancer Genome Atlas gene expression was also analysed on pretreatment tumor tissue: responses were more frequent in the Basal I subtype according to Atlas classification, which showed the strongest association with interferon-y signature and the highest CD8 expression [31] (Table 1).

At 2017 ASCO Genitourinary Cancer Symposium, preliminary data about combination of nivolumab and ipilimumab have been presented. Ten patients with advanced or metastatic urothelial cancer, refractory to nivolumab monotherapy, were treated. Despite a slight increase of immune-related toxicities, treatment was well tolerated and showed a promising activity, with a disease control rate of 40% (one partial response and three stable disease were reported) [32]. Of course, the number of patients described in this preliminary experience is still too small to comment the activity of the combination. Trials ongoing evaluating nivolumab in combination with ipilimumab are shown in Table 3 and will clarify the real potential of the immune-therapy combination.

### 4.2. Pembrolizumab

Pembrolizumab is a humanized monoclonal antibody directed against PD-1, which has shown promising results for treatment of metastatic bladder cancer. Results about urothelial cancer patients' cohort of the nonrandomized, multicohort, open-label, phase 1b Keynote 012 basket trial were published in January 2017. Thirty-three patients with advanced or metastatic urothelial cancer with at least 1% PD-L1 expression in tumor cells or stroma were enrolled and treated with 10 mg/kg intravenous pembrolizumab every two weeks, until progressive disease or unacceptable toxicity. Treatment was generally well tolerated, and only 9% of patients experienced serious adverse events. Seven of 27 evaluable patients (26.0%; 95% CI, 11.0–46.0) achieved partial or completed responses [33] (Table 1).

At 2017 ASCO Genitourinary Cancer Symposium, preliminary data of phase II Keynote 052 trial have been presented. In detail, this trial evaluated activity and safety of pembrolizumab in cisplatin-ineligible patients with metastatic or locally advanced bladder cancer, enrolled regardless of PD-L1 expression. However, PD-L1 expression was prospectively assessed in tumor and immune cells, to better characterize responding and nonresponding patients. Patients received pembrolizumab 200 mg intravenously every three weeks, for up to 24 months of treatment. Among patients with at least four months of follow-up, ORR was 27% (95% CI, 22–32); no data about activity according to PD-L1 expression were reported. The only data available have been presented at ESMO 2016 Congress: ORR was 36.7% (95% CI, 19.9–56.1) in patients with 10% or greater PD-L1 expression [34, 35] (Table 1).

Of note, a randomized phase III trial, Keynote 045 study, compared pembrolizumab to chemotherapy (consisting of either paclitaxel, docetaxel, or vinflunine according to Investigator's choice) in 542 patients with locally advanced unresectable or metastatic urothelial carcinoma recurring or progressing following platinum-based chemotherapy. A survival benefit was shown in the pembrolizumab group (median OS was 10.3 versus 7.4 months, hazard ratio for death, 0.73; 95% CI, 0.59 to 0.91; *p* = 0.002), regardless of PD-L1 expression; also ORR was significantly improved in the pembrolizumab group (21.1% versus 11.4%; *p* = 0.001). These benefits were similar across almost all subgroups examined, regardless of the type of chemotherapy or the presence of poor prognostic factors, such as hepatic metastases. Fewer adverse events for any grade occurred in the pembrolizumab group compared to patients treated with chemotherapy (Table 1) [36].

Various studies are ongoing investigating pembrolizumab activity in combination with other systemic therapies and radiotherapy (Table 2).

## 5. Drugs That Target CTLA-4

### 5.1. Ipilimumab and Tremelimumab

Safety and immunologic pharmacodynamic effects of ipilimumab, an anti-CTLA-4 monoclonal antibody, have already been evaluated in the neoadjuvant setting in a small phase II clinical trial. Twelve patients with localized, high grade, urothelial carcinoma of the bladder were treated with ipilimumab, at the dose of 3 or 10 mg/kg. Safety profile of treatment was good. In all patients, an increase in CD4 + T-cell population in both tumor tissue and peripheral blood was found, probably positively related to clinical benefit. Of note, eight patients showed tumor regression: on radical cystectomy specimens, obtained after neoadjuvant treatment, lower stages of disease were found [37].

Several trials are now ongoing, to evaluate anti CTLA-4 antibody ipilimumab or tremelimumab, alone or in combination with nivolumab or durvalumab or chemotherapy or other target therapies (Table 3). Results are not yet available.

## 6. Other Immunotherapies

Immunotherapy includes treatments that work in different ways, not only limited to anti-PD-1, anti-PD-L1, or anti-CTL A4 antibodies. There are many potential targets under study: antigens on tumor cells surface, new immune-checkpoints, and tumor microenvironment. Against some of these targets, vaccines and monoclonal antibody are on development, even if few results from clinical trials are available at the time.

### 6.1. Immune System Targets

#### 6.1.1. Recombinant Interleukin-2

One of the first attempts of immunotherapy foresaw the use of recombinant interleukin-2 (rIL-2), a cytokine whose main function is to promote T-cell differentiation and activation. In 1991, nine patients with metastatic transitional bladder cancer were treated with a continuous infusion of rIL-2 associated with lymphocytes previously stimulated in vitro with the same cytokine. Unfortunately none of the patients benefited from that treatment: at the first radiological evaluation eight patients showed progression disease and one patient had stable disease [18].

#### 6.1.2. ALT-801

More recently at ASCO 2015 annual meeting, preliminary results of a phase Ib/II study of cisplatin and gemcitabine in combination with ALT-801, an IL-2/T-cell receptor fusion protein, in advanced or metastatic urothelial carcinoma were presented. Dose escalation expansion cohort phase Ib study included both chemonaïve and chemorefractory patients (group 1), whereas phase II expansion study included only chemorefractory patients (group 2). 34 of the 62 enrolled patients were chemorefractory. Among these patients, ORR was 35% (95% CI: 20–54%), and median OS was 12.3 months for group 1 (data not available for group 2 and for chemonaïve patients). Almost all patients experienced severe hematological toxicities [38].

#### 6.1.3. B7-H3

B7H3, also known as CD276, is a ligand of the B7 family, which also includes the better known PD-1 and PD-L1. Even if its receptor remains unidentified, B7H3 acts as coinhibitor of peripheral immune response, and its expression seems to be particularly intense in urothelial carcinoma and could correlate with poor prognosis [39]. A dose escalation phase I trial is ongoing (NCT01391143) to evaluate toxicity and potential antitumor activity of the monoclonal antibody MGA271 (enoblituzumab), in patients with various refractory cancers, including urothelial cancer that express B7H3 antigen. Preliminary data were presented at the 2015 Society for Immunotherapy of Cancer (SITC) Annual Meeting. Treatment showed an optimal tolerability with few severe adverse events and a promising activity in patients with melanoma, prostate, and bladder cancer [40].

Another phase I trial (NCT02628535) is currently recruiting participants to assess safety and establish the maximum tolerated dose (MTD) of MGD009, a humanized, Dual-Affinity Retargeting, or DART® molecule that recognizes both B7-H3 and CD3. Patients must have B7-H3 positive unresectable locally advanced or metastatic tumors, including bladder cancer.

#### 6.1.4. OX-40 and 4-1bb

OX-40 and 4-1bb, also known respectively as CD134 and CD137, are both members of the Tumor Necrosis Factor receptor (TNF-r) super-family. The former is expressed on CD4 and CD8 T-cell surfaces, the later on NK and activated T-cells. The activation of both signal pathways promotes T-cell proliferation and survival. Moreover OX-40 provides a stimulatory signal to effectors and memory T-cell population, and an inhibitory signal to regulatory T-cells [41, 42].

A phase I dose escalation study (NCT02315066) is currently recruiting participants to assess safety and potential activity of an experimental OX-40 agonist alone or in combination with a 4-1bb agonist, in patients with various tumors, including urothelial bladder carcinoma.

#### 6.1.5. TILs

Another promising therapeutic strategy is the infusion of externally manipulated T-cells that could be extracted from tumor tissue, the so-called tumor infiltrating lymphocytes or TILs, to give rapid immunity. TILs could be simple expanded ex vivo or selected according to recognized antigens. In a small open trial twelve patients underwent surgery for stage IV bladder cancer and TILs from lymph nodes draining metastatic tumors were collected. In six of them, lymphocytes were infused after in vitro expansion without any severe AEs. No data are available on the efficacy of the treatment [43]. A phase II trial (NCT01174121) is now recruiting patients with metastatic solid tumors, including urothelial bladder cancer, with at least one resectable lesion for TILs generation. Lymphocytes will be reinfused after conditioning chemotherapy and pembrolizumab administration. Results of another trial (NCT02863913), not yet open for participants' recruitment, will add important evidence. It is a phase I dose escalation clinical trial to assess the safety of PD-1 knockout engineered T-cells in treating metastatic advanced bladder cancer.

### 6.2. Tumor Targets

#### 6.2.1. HER2

Human epidermal growth factor receptor 2 (HER2), also known as CD340, is a member of a big receptor family, encoded by a protooncogene whose alterations (almost amplification and overexpression) are common not only in breast and gastric, but also in urothelial cancer. HER2 target therapy had shown interesting activity in preclinical studies and phase I clinical trials. Unfortunately no difference in efficacy on addiction of trastuzumab to standard chemotherapy with platinum and gemcitabine was detected in advanced or metastatic urothelial carcinoma overexpressing HER2 in a phase II clinical trial [44]. At 2017 ASCO Genitourinary Cancer Symposium preliminary results of the ongoing phase IIA MyPathway trial were presented. Twelve patients with platinum-resistant HER2-positive metastatic urothelial cancer have been enrolled, and at a median follow-up of 5.4 months there were a CR, two PR, and two stabilisation of disease [45]. Other clinical trials are still ongoing in these patients, testing other HER2 inhibitors, like trastuzumab emtansine (NCT02999672) and Lapatinib (NCT00949455, NCT02342587).

Alternative strategies are under development, looking at HER-2 as a target for immunotherapy. A dendritic cell vaccine called AdHER2, created using an individual's own immune cells, has been developed to stimulate the immune system to recognize HER-2. A phase I study (NCT01730118) is now recruiting patients with various solid tumors and HER2 overexpression [46].

#### 6.2.2. hCG-*β*

Human Chorionic Gonadotropin beta-chain (hCG-*β*) is an antigen frequently expressed by epithelial malignancies, including urothelial cancer. Elevated hCG-*β* serum levels and/or tissue expression are associated with a more aggressive disease course. CDX-1307 is a human monoclonal antibody against the APC mannose receptor fused to hCG-*β* that acts like a vaccine. Internalized by APCs, CDX-1307 is processed and hCG-*β* is presented as an antigen, inducing specific cellular and humoral immune response.

A phase I trial demonstrated that CDX-1307 is well tolerated and active, inducing consistent humoral and T-cell responses when coadministrated with Granulocyte-Macrophage Colony-Stimulating Factor (GM-CSF) and Tall Like Receptor (TLR) agonists, in patients with advanced epithelial malignancies, including bladder cancer [47]. A phase II trial (NCT01094496) to evaluate antitumor activity before and after bladder surgery has recently been completed, but results are not available.

#### 6.2.3. MAGE-A

Melanoma associated antigen A (MAGE-A) are a family of tumor specific antigens expressed in several cancer cell types, but not in normal tissue, except for the testis. MAGE-A proteins are recognized by cytotoxic T-cells and are promising targets for immunotherapy [48]. Partial or complete responses after MAGE-A target immunotherapy have been reported, also for advanced bladder cancer. Particularly three of four heavily pretreated patients with high expression of MAGE-A were enrolled in a small pilot clinical trial. They were treated with subcutaneous injections of autologous dendritic cells pulsed with MAGE-A3 epitopes peptides, showing significant reduction of tumor burden [49].

In a phase I/early II trial, patients with stage III or IV malignancies, including three with bladder cancer, all MAGE-3 positive, were randomized to three different escalation dose levels of a recombinant MAGE-3 vaccine, associated with fixed doses of an immunological adjuvant, in order to further improve its immunogenicity. One of the bladder cancer patients showed a short-term almost complete response of two months [50].

A dose escalation phase I trial (NCT02989064) is now recruiting patients with MAGE-410 positive advanced malignancies, including bladder cancer. Treatment protocol provides the administration of autologous genetically modified MAGE A10 T-cells, and the primary endpoint is the evaluation of safety and tolerability of that treatment.

#### 6.2.4. NY-ESO-1

NY-ESO-1 is one of the most immunogenic tumor antigens, expressed in cancer and testis, but not in other normal tissues (similarly to MAGE-A1). It is expressed in approximately 25–30% of bladder cancers, highly in advanced stages, and it is considered one of the best targets for T-cell receptor (TCR) based immunotherapy in solid cancers. NY-ESO-1-specific T-cell responses, induced in cancer patients using NY-ESO-1 peptides, proteins, and viruses encoding NY-ESO-1, are too weak to eradicate tumor cells [51]. So to improve clinical response TCR gene therapy is being developed. Two phase I trials (NCT02457650 and NCT02869217) are currently recruiting participants with NY-ESO-1-expressing malignancies to evaluate the safety and feasibility of the administration of anti-NY-ESO-1 TCR engineered autologous T-cells.

### 6.3. Peptide Personalized Vaccination

In the context of an increasingly personalized medicine, an open-label, randomized phase II trial evaluated safety and efficacy of peptide personalized vaccination compared to best supportive care in eighty patients with advanced urothelial bladder cancer progressing after platinum-containing chemotherapy. Vaccination consisted in subcutaneous injections of maximum four peptides chosen from a pool of thirty-one peptides according to patients HLA type and specific peptide-reactive IgG titers. PR was observed in 9 (23%) patients in the experimental arm, with mo CR. A significant improvement in OS was also noted (HR, 0.58; 95% CI, 0.34–0.99, *p* = 0.049), but not in PFS. Treatment was fairly well tolerated; almost all AEs were of grade 1 or 2; there were no grade 4 AEs or treatment-related deaths. Obviously, as the authors concluded, further large-scale, randomized trials are needed to confirm these results [52].

## 7. Discussion

The encouraging results recently obtained with several immune checkpoint inhibitors [23–25, 27, 31, 36] raise enthusiasm about the future role of this therapeutic approach for patients affected by advanced urothelial cancer. As well known, standard treatment for these patients is platinum-based chemotherapy, characterized by a difficult balance between efficacy and treatment toxicity. The availability of immune checkpoint inhibitors, both in patients who are considered medically unfit for cisplatin and in patients who have experienced disease progression after chemotherapy, represents a clinically valuable opportunity. Interestingly, a nonnegligible proportion of patients experience a durable disease control, with a chance of long-survival that has been observed, with the use of these drugs, in many types of solid tumors.

However, similar to what is occurring also in other tumors, knowledge about predictive factors of efficacy and patients' selection criteria for immunotherapy is still not ideal and, within all the clinical trials, a relevant number of patients failed to respond to the PD-1/PD-L1 checkpoint blockades. From this point of view, it would be crucial to identify a biomarker to predict the response to checkpoint blockades. In principle, a perfect positive predictive value could allow avoiding treating patients who are not going to obtain any benefit, while a perfect negative predictive value could allow not denying treatment to any of the patients who could potentially benefit. Unfortunately, at the moment, we have no biomarker with a good positive and negative predictive value. The expression of PD-L1 has been studied as a putative biomarker in many of the trials testing anti-PD-1 and anti-PD-L1 drugs, but PD-L1 staining cannot be used to accurately select patients for PD-1/PD-L1 pathway blockade due to the low prediction accuracy and dynamic changes [53]. Interestingly, tumor infiltrating immune cells and molecules in the tumor microenvironment, alone or along with PD-L1 expression, could be useful as predictive factors [53]. Furthermore, gene analysis (gene signatures, mutational landscape, and/or mismatch-repair deficiency) could be useful if interesting preliminary evidence will be confirmed and validated in further studies [23, 24].

The diffusion of immune checkpoint inhibitors in clinical practice will imply the confidence of medical oncologists with the diagnosis and management of typical side effects associated with this therapeutic approach [54].

As for the applicability of trials results in clinical practice, reasonably, we will have no direct comparisons between different anti-PD-1 and anti-PD-L1 that are currently undergoing the clinical development. In the absence of obvious differences in efficacy emerged in indirect comparison of clinical trials, we do not know which is the best treatment choice. Other issues that are not completely answered by the evidence produced in clinical trials are the dose-response relationship (recent evidence in melanoma with ipilimumab suggests that higher dose is associated with higher efficacy [55]) and the optimal duration of treatment (continuous until disease progression or with planned “stop-and-go”).

Currently ongoing trials will clarify the role of immune checkpoint agents as first-line treatment, compared to platinum-based chemotherapy. Based on the results of these trials, along with the trials testing other categories of immune treatments, treatment paradigm for patients affected by advanced urothelial cancer could be soon radically changed compared to current guidelines.

## Figures and Tables

**Table 1:** Clinical trials with anti-PD-L1 and anti-PD-1 immune checkpoint inhibitors in metastatic urothelial cancer.

Drug	Trial	Phase	Indication	Sample size (*n*)	Control arm	Results in all pts	Results according to PDL1
Atezolizumab 1200 mg IV q3w^*∗*^	ImVigor 210	II	Cohort A: 1° line, cisplatin ineligible [24]	123		**IR-ORR** 23% (95% CI 16–31) *IA-ORR:* 25% (18–34) *DOR* not reached (range 3.7–21.0+) *PFS* 2.7 months (95% CI 2.1–4.2) *OS* 15.9 m (95% CI 10.4 to N.E.)	**IR-ORR** 28% (14–47) in IC2/3, 24% (15–35) in IC1/2/3, 21% (95% CI 10–35) in IC1, and 21% (95% CI 9–36) in IC0 *IA-ORR:* 31% (16–36) in IC2/3, 25% (16–36) in IC1/2/3, 21% (95% CI 11–35) in IC1, and 26% (95% CI 13–42) in IC0 *PFS* 4.1 m (2.3–11.8) in IC2/3, 2.1 m (2.1–5.4) in IC1, and 2.6 m (2.1–5.7) in IC0 *OS* 12.3 m (6.0 to N.E.) in IC2/3, and 19.1 m (9.8 to N.E.) in IC0/1
			Cohort B: postplatinum [23]	310		**IR-ORR**: 15% (95% CI 11–20) **IA-ORR**: 19% (95% CI 15–24) *DOR:* not reached (range 2.0–13.7 m) *IR-PFS:* 2.1 m (95% CI 2.1-2.1) *IA-PFS:* 2.7 m (95% CI 2.1–3.9) *OS:* 7.9 m (95% CI 6.6–9.3) *OS at 12 m:* 36% (95% CI 30–41) * Safety:* Any grade TRAE 215 (69%), serious TRAE 50 (16%)	**IR-ORR**: 26% (95% CI 18–36) in IC2/3, 18% (95% CI 13–24) in IC1/2/3 **IR-ORR**: 27% (95% CI 19–37) in IC2/3, 22% (95% CI 16–28) in IC1/2/3 *DOR:* *IC-PFS:* 2.1 m (95% CI 2.1–4.1) in IC2/3, 2.1 m (95% CI 2.1-2.1) in IC1/2/3 *IA-PFS:* 4.0 m (95% CI 2.6–5.9) in IC2/3, 2.9 m (95% CI 2.1–4.1) in IC1/2/3 *OS:* 11.4 m (95% CI 9.0–N.E.) in IC2/3, 8.8 m (95% CI 7.1–10.6) in IC1/2/3 *OS at 12 m:* 48% (95% CI 38–58) in IC2/3, 39% (95% CI 32–46) in IC1/2/3

Durvalumab 10 mg/kg q2w [25]	NCT01693562.	I/II	Unresectable or metastatic	61		**Safety**: Any grade AE 39 (63.9%), serious AE 3 (4.9%) *IA-ORR* 31.0% (95% CI, 17.6 to 47.1) in all pts	*IA-ORR:* 46.4% in PD-L1+ and 0% in PD-L1− *DCR at 12 w:* 57.1% in PD-L1+ and 28.6% in PD-L1−

Avelumab 10 mg/kg q2w [27]	NCT01772004	I	Postplatinum or cisplatin ineligible	44		**Safety**: Any grade AE 29 (65.9%), serious AE 3 (6.8%) *IR-ORR:* 18.2% (95% CI, 8.2%–32.7%) *OS:* 13.7 m (95% CI, 8.5–N.E.) *PFS:* 11.6 w (95% CI, 6.1–17.4)	*ORR:* 53.8% in PD-L1 ≥ 5% versus 4.2% in PD-l1 < 5%

Nivolumab 3 mg/kg IV q2w	Checkmate 032 NCT01928394. [30]	I/II	Postplatinum or refusing it	78		**IA-ORR**: 19 (24.4%, 95% CI 15.3–35.4) *DOR:* 1.5 m (1.2–4.1) *PFS:* 2.8 m (95% CI 1.5–5.9) *OS:* 9.7 m (95% CI 7.3–16.2) *Safety:* Any grade TRAE 63 (81%), serious TRAE 17 (22%)	**IA-ORR**: 24% (95% CI 9–45) in PD-L1 ≥ 1%, 26% (14–42) in PD-L1 < 1%, 24.5 (13.8–38.3) in PD-L1 < 5%, 28.6 (8.4–58.1) in PD-L1 ≥ 5% *PFS:* 5.5 m (95% CI 1.4–11.2) in PD-L1 ≥ 1%, 2.8 m (1.4–6.5) in PD-L1 < 1%, 2.8 (1.5–7.0) in PD-L1 < 5%, 5.5 (1.2–11.2) in PD-L1 ≥ 5% *OS:* 16.2 m (95% CI 7.6–N.E.) in PD-L1 ≥ 1%, and 9.9 m (7.0–N.E.) in PD-L1 < 1%, 10.4 (7.0–N.E.) in PD-L1 < 5%, 12.9 (2.8–N.E.) in PD-L1 ≥ 5%
	Checkmate 275 NCT02387996 [31]	II	Postplatinum (no liver metastasis if ≥2 CT lines)	270		**IR-ORR**: 19.6% (95% CI 15.0–24.9) *IA-ORR:* N.A. *PFS:* 2.00 m (95% CI 1.87–2.63) *OS:* 8.74 m (95% CI 6.05–N.E.)	**IR-ORR:** 28.4% (95% CI 18.9–39.5) in PD-L1 ≥ 5%, 23.8% (95% CI 16.5–32.3) in PD-L1 ≥ 1%, 16.1% (95% CI 10.5–23.1) in PD-L1 < 1% *IA-ORR:* N.A. *PFS:* N.A. *OS:* 11.30 m (8.74–N.E) in PD-L1 ≥ 1%, 5.95 m (4.30–8.08) in PD-L1 < 1%

Nivo 240 mg q2w → Nivo 3 mg/kg + Ipi 1 mg/kg q3w, then Nivo 240 mg q2w	CA209- 260 NCT02553642 [32]	2	Metastatic, option of treatment with the combination if confirmed PD with Nivo	40 (10 treated with Nivo + Ipi)		**DCR**: 4/10 (1 partial response and 3 stable diseases)	

Pembrolizumab 200 mg q3w	Keynote 012 NCT01848834 [33]	Ib	Unresectable or metastatic	33		**IR-ORR**: 26% (95% CI 11–46) **Safety**: Any grade TRAE 20 (61%), serious TRAE 5 (15%) *IA-ORR:* *PFS:* 2 m (95% CI 2–4) *DOR:* 10 m (range 4–22+) *OS:* 13 m (95% CI 5–20)	Patients were required to have ≥1% PD-L1 expression
	Keynote 052 NCT02335424 [34]	II	Cisplatin ineligible	370		**IR-ORR**: 27% (22–32) *DOR:* (1+ to 14+ m) *PFS 6 m:* 31% *OS 6 m:* 67%	
	Keynote 045 NCT02256436 [36]	III	Postplatinum	542	Investigator's choice CT with paclitaxel, docetaxel, or vinflunine	**OS**: 10.3 versus 7.4 m (HR: 0.73; *p* = 0.002) **PFS**: 2.1 versus 3.m (HR: 0.98; *p* = 0.42) *ORR:* 21.1 versus 11.4% (*p* = 0.001) *DOR:* not reached versus 4.3 m	**OS**: 8 versus 5.2 (HR: 0.57; *p* = 0.005) in PD-L1 ≥ 10% **PFS**: HR: 0.89 *p* = 0.24 *ORR:* N.A. *DOR:* N.A.

AE: adverse events, CT: chemotherapy, DOR: duration of response, IA: investigator-assessed, IC: immune cells, IR: independently reviewed, m: months, N.A.: not available, N.E.: not estimable, ORR: overall response rate, OS: overall survival, PFS: progression free survival, TRAE: treatment-related adverse events, w: weeks; bold refers to primary endpoint; italics refers to secondary endpoint; ^*∗*^PD-L1 expression status was defined by the percentage of PD-L1+ IC: IC0 (<1%), IC1 (≥1% but <5%), and IC2/3 (≥5%).

**Table 2:** Ongoing clinical trials of anti PD-L1 and anti PD-1 immune checkpoint inhibitors in metastatic urothelial cancer.

	Study	Phase	Regimen	Primary endpoints	Planned number of pts or pts enrolled	Status
Atezolizumab	NCT02302807 (IMVigor 211)	III	Atz 1200 mg IV d1 q3w versus CT (Vnf 320 mg/m^2^ or Txl 175 mg/m^2^, or Txt 75 mg/m^2^) IV d1 q3w	OS	932	Active, not recruiting
	NCT02807636 (IMVigor 130)	III	Atz 1200 mg IV d1 + CT (Crb AUC 4.5 IV d1 + Gem 1000 mg/m^2^ IV d1,8 q3w) versus Placebo + CT	OS, PFS and Safety	435	Currently recruiting
	NCT02989584	II	Atz 1200 mg IV d8 q3w + Gem 1000 mg/m^2^ IV d1,8 + Cis 70 mg/m^2^ d1 q3w (maintenance in phase II)	Safety	30	Currently recruiting
	NCT02298153 (ECHO-110)	I	Atz 1200 mg IV q3w + Epacadostat 25 mg OS BID as starting dose, followed by dose escalations.	Safety	118	Currently recruiting
	NCT02928406	III	Atz 1200 mg IV q3w	Safety	1000	Active, not recruiting
	NCT02655822	I	CPI-444 in 3 different schedules versus CPI-444 + Atz IV	Safety, ORR, median AUC of CPI-444	534	Currently recruiting
	NCT02543645	I/II	Varlilumab 0.3 or 1 or 3 mg/kg + Atz 1200 mg IV q2w	Safety, ORR	55	Currently recruiting

Durvalumab	NCT02516241	III	IV Drv +/− IV Trm versus CT (platinum + Gem)	PFS, OS	1005	Active, not recruiting
	NCT02546661 (Biscay)	I	(A) Drv + AZD4547 (B) Drv + olaparib (C) Drv + AZD1775 (D) Drv (E) Drv + Vistusertib	Safety	110	Currently recruiting
	NCT02527434	II	IV Trm versus IV Trm + IV Drv versus IV Drv	ORR	66	Currently recruiting
	NCT02643303	I/II	IV Drv + IV Trm +/− IT/IM PolyICLC	Recommended combination dose, safety, ORR, PFS and OS	102	Active, not recruiting
	NCT02318277	I/II	Drv IV q2w + OS INCB024360 25 mg BID followed by dose escalations.	DLT, ORR	185	Currently recruiting

Avelumab	NCT02603432 (JAVELIN Bladder 100)	III	Avl IV q2w + BSC versus BSC	OS	668	Currently recruiting

Nivolumab	NCT02387996	II	IV Niv	ORR	242	Active, not recruiting
	NCT02897765	I	Niv IV 240 mg q2w +/− NEO-PV-01 SC + Adj	Safety	90	Currently recruiting
	NCT02496208	I	OS cabozantinib-s-malate + IV Niv +/− IV Ipi	Safety and DLT	66	Currently recruiting
	NCT01928394 (Checkmate 032)	I/II	IV Niv +/− IV Ipi (different schedules) +/− OS Cobimetinib	ORR	1150	Currently recruiting
	NCT02636036 (SPICE)	I	IV Niv + IV Enadenotucirev	MTD	30	Currently recruiting
	NCT02834013 (DART)	II	Niv IV d1,15,29 + Ipi IV d1 q6w	ORR	334	Active, not recruiting
	NCT02614456	I	Phase 1: IFN-*γ* SC 50 *μ*g/m^2^ d1–7 Phase 2: IFN-*γ* SC QD + Niv IV d1 q2w Phase 3: Niv IV d1 q3w	Safety, DLT	15	Currently recruiting

Pembrolizumab	NCT02717156	II	Pmb IV d1 + EphB4-HSA IV d1,8,15 q3w	Safety	60	Active, not recruiting
	NCT02925533	I	IV B-701 + IV Pmb q3w	Safety	12	Currently recruiting
	NCT02560636 (PLUMMB)	I	IV Pmb + RT	MTD, Safety	34	Currently recruiting
	NCT02351739 (Keynote 143)	II	IV Pmb +/− ACP-196	ORR	75	Active, not recruiting
	NCT02500121	II	Pmb 200 mg IV d1 q3w versus placebo	6 months PFS	200	Currently recruiting
	NCT02853305 (Keynote 361)	III	Pmb 200 mg IV d1 q3w +/− CT versus CT (platinum + Gem)	PFS, OS	990	Currently recruiting
	NCT02619253	I/II	Pmb 200 mg IV d1 q3w + Vorinostat OS d1–14 q3w	Safety	42	Currently recruiting
	NCT02826564	I	Stereotactic body radiotherapy prior to or concurrent with IV Pmb	Safety, selection of the sequence arm with a DLT < 20%	20	Currently recruiting
	NCT02880345 (Radvax)	Pilot	IV Pmb + hypofractionated RT (2 different regimens)	Safety	14	Active, not recruiting
	NCT02437370	I	IV Pmb + IV Txt versus IV Pmb versus IV Gem	MTD	38	Currently recruiting
	NCT02043665 (Keynote 200)	I	(A) CVA21 (B) CVA21 + Pmb	ORR	60	Currently recruiting
	NCT02581982	II	Pmb 200 mg IV d1 + Txl IV d1,8 q3w	ORR	27	Currently recruiting
	NCT01174121	II	Cyclophosphamide and fludarabine + Pmb + young TIL	Rate of tumor regression	290	Currently recruiting
	NCT03006887	I	Pmb 200 mg IV d1 + Lenvatinib OS 20 mg QD q3w	Safety, DLT	10	Active, not recruiting
	NCT02501096	I/II	Pmb 200 mg IV d1 + Lenvatinib OS QD q3w	MTD, ORR, DLT	250	Currently recruiting
	NCT02346955 (MK-6018-001)	I	Multidose escalation of CM-24 +/− Pmb 200 mg IV	Safety, DLT	196	Currently recruiting
	NCT02452424	I/II	Dose escalation of OS PLX3397 + Pmb 200 mg IV	Safety	400	Currently recruiting
	NCT02432963	I	IV Pmb + SC MVA-p53 Vaccine	Tolerability	19	Currently recruiting
	NCT02393248	I/II	Phase 1: dose escalation/expansion of INCB054828 Phase 2: INCB054828 + Pmb/CT (Txt or Cis + Gem)	MTD, pharmacodynamics	150	Currently recruiting
	NCT02443324	I	IV Pmb + Ramucirumab IV d1 q3w	DLT	155	Currently recruiting
	NCT02856425	I	IV Pmb + OS Nintedanib	MTD	18	Currently recruiting

Atz: atezolizumab; Avl: avelumab; Cis: cisplatin; Drv: durvalumab; Gem: gemcitabine Ipi: ipilimumab; Trm: tremelimumab; Txl: taxol; Txt: taxotere; Niv: nivolumab; Pmb: pembrolizumab.

**Table 3:** Ongoing clinical trials of anti-CTLA-4 immune checkpoint inhibitors in metastatic urothelial cancer.

	Study	Phase	Regimen	Primary endpoints	Planned number of pts or pts enrolled	Status
Ipilimumab	NCT01524991	II	IV gemcitabine 1000 mg/m^2^ d 1,8 + cisplatin 70 mg/m^2^ d1 q3w. IV Ipi 10 mg/kg d1 (start c3)	1 year OS	36	Active, not recruiting
	NCT02496208	I	OS cabozantinib-s-malate + IV Niv +/− Ipi	Safety and DLT	66	Currently recruiting
	NCT01928394	I/II	IV Niv +/− Ipi (different schedules) +/− cobimetinib	ORR	1150	Currently recruiting
	NCT02381314	I	IV Ipi d1 q3w + IV enoblituzumab weekly	Safety	84	Currently recruiting
	NCT02834013 (DART)	II	IV Niv d 1,15,29 + IV ipilimumab d1 q6w	ORR	334	Active, not recruiting

Tremelimumab	NCT02516241	III	IV Drv +/− IV Trm versus CT (platinum + gemcitabine)	PFS, OS	1005	Active, not recruiting
	NCT02527434	II	IV Trm versus IV Trm + IV Drv versus IV Drv	ORR	66	Currently recruiting
	NCT02643303	I/II	IV Drv + IV tremelimumab +/− IT/IM PolyICLC	Recommended combination dose, safety, ORR, PFS, and OS	102	Active, not recruiting
